# Auditory processing and communication in autism: exploring verbal abilities and vocal affective cues

**DOI:** 10.3389/fpsyt.2026.1754358

**Published:** 2026-04-23

**Authors:** Alec Gallo, Jennifer Henderson Sabes, Carly Demopoulos

**Affiliations:** 1 Department of Psychiatry and Behavioral Sciences, University of California, San Francisco, San Francisco, CA, United States; 2 School of Medicine, University of California, San Francisco, San Francisco, CA, United States; 3 Department of Radiology and Biomedical Imaging, University of California, San Francisco, San Francisco, CA, United States

**Keywords:** auditory processing, autism, children and adolescents, sensory processing, verbal abilities, vocal affect recognition

## Abstract

This study examined the role of auditory processing in autism spectrum disorder, focusing on its association with verbal and non-verbal vocal communication skills in children and adolescents. A total of 97 English-speaking autistic participants (ages 7.9–17.4 years, mean = 12.3) and 44 neurotypical peers (ages 8.4-16.8, mean= 12.3) completed assessments of auditory processing and communication skills. We analyzed the relationships between scores on the SCAN-3 Tests for Auditory Processing Disorders time-compressed sentences, auditory figure-ground (+ 8dB), gap detection, and competing words-free recall subtests, the Clinical Evaluation of Language Fundamentals-Fifth Edition (CELF-5) expressive and receptive language indices, the Goldman-Fristoe Test of Articulation-3rd Edition (GFTA-3) Sounds-In-Words subtest, and the Diagnostic Analysis of Nonverbal Accuracy-2nd Edition (DANVA-2) paralanguage subtests. Measures of auditory processing were associated with both verbal and non-verbal communication skills in the autistic participants. Specifically, we found that SCAN-3 time-compressed sentence and gap detection scores were associated with expressive and receptive language skills, receptive vocabulary scores, and ability to recognize vocal emotional cues. Gap detection abilities additionally correlated positively with expressive and receptive language skills, while auditory figure-ground task performance was related to articulation. In conclusion, this study suggests specific aspects of auditory processing may be important for development of specific aspects of auditory communication skills in children on the spectrum. Specifically, spectral aspects of auditory processing abilities were associated with articulation accuracy whereas temporal components of auditory processing may impact broader verbal and nonverbal communication skills. Further research is needed to better understand the underlying mechanisms of these associations and potential directions of causality to inform development of interventions that target auditory processing and auditory communication skills in ASD.

## 1 Introduction

The ability to process rapidly presented auditory stimuli is thought to be fundamental for successful language development (1, 2). Individuals with developmental language disorders, and those on the autism spectrum, often exhibit difficulties in auditory processing. Autism spectrum disorder (ASD) affects approximately 1 in 100 children worldwide and is frequently accompanied by clinically significant communication difficulties that persist across development and substantially impact academic, social, and adaptive functioning (3–5). While auditory processing difficulties have been repeatedly linked to communication impairments, prior studies have focused on either verbal or non-verbal domains separately or have examined a single aspect of auditory processing. This study is the first to examine associations between multiple domains of auditory processing and both verbal and nonverbal auditory communication skills in autistic and neurotypical youth. The findings can contribute to a more detailed understanding of the mechanisms underlying communication difficulties in autism and support the development of targeted approaches to improve communication. By integrating auditory processing measures with clinically relevant communication outcomes, this work aims to clarify whether specific auditory mechanisms may represent potentially meaningful targets for assessment and intervention.

Verbal and non-verbal communication impairments are core features of autism, affecting a range of linguistic and social communicative abilities (3, 6, 7). Many autistic individuals exhibit challenges in receptive and expressive language, including deficits in phonology, morphology, syntax, pragmatics, and semantics, in both oral and written language domains (8–10). Clinically, language ability is one of the strongest predictors of long-term functional outcomes in ASD, including academic achievement, employment, and independent living, underscoring the importance of identifying mechanistic contributors to language variability within the spectrum (7). Hudry et al. (11) observed lower performances in language comprehension abilities among ASD preschoolers compared to their age expectations, with receptive language development often trailing behind the development of expressive language abilities compared to neurotypical peers. However, other studies failed to replicate such findings (12–14). Differences in vocabulary scores are also notable in domains that require understanding one’s own or others’ emotional and cognitive states. For example, (15) reported differences in emotion-related vocabulary, suggesting challenges in mapping words to internal affective states; while (16, 17) found difficulties in words that convey perspective and mental state, such as personal pronouns, mental state terms, and prepositions.

Furthermore, phonology in autistic children shows variation, with some children exhibiting differences in phonology and speech sound production, particularly those who require more support (16, 18). While some studies have not found articulatory problems across diverse age groups and different tasks, including the Reynell Developmental Language Scales and Goldman-Fristoe Test of Articulation (GFTA) (13, 19), autistic children with lower vocabulary scores, measured by the Peabody Picture Vocabulary Test-Fifth Edition (PPVT-5) and the Clinical Evaluation of Language Fundamentals-Third Edition (CELF-3), showed differences on the pseudoword repetition test (13). Together, this evidence highlights the wide range of variability in verbal communication skills in the autism population.

Children on the spectrum also face challenges in non-verbal vocal communication. Non-verbal communication is a broad construct that includes information conveyed without words across multiple modalities, such as facial expressions, gestures, and body posture. In contrast to verbal communication, non-verbal vocal communication refers specifically to paralinguistic information carried in the voice, such as prosody, pitch, rhythm, and intensity, that conveys affective and social meaning independent of lexical content. Tasks that assess vocal affect recognition (e.g., identifying emotion from semantically neutral sentences spoken with different intonations) therefore rely heavily on the perception and integration of fine-grained acoustic cues over time. Distinguishing these constructs is important because non-verbal vocal communication places particularly high demands on auditory temporal and spectral processing, which are domains frequently reported as atypical in autism. Several studies have identified differences in how individuals with ASD interpret vocal affect (20–27). Vocal affect recognition requires the integration of multiple auditory cues, including variations in pitch, volume, speech quality, and the speed of utterance over time (28), with social contextual information to form an affective interpretation (29), making it a particularly demanding process for individuals on the spectrum. Therefore, successful extraction of emotional meaning from speech depends on both higher-order social cognition, and on intact processing of low-level acoustic features over time. Deficits in processing basic auditory cues, such as pitch and temporal changes, have been associated with difficulties in extracting socially relevant information from speech, which may further exacerbate communication challenges in ASD (30). These difficulties in vocal affect recognition have been associated with poor social skills (31) and autistic traits (30). These findings indicate that vocal emotion recognition difficulties in ASD may arise from higher-level social cognitive processes as well as atypical processing of basic auditory features of others’ vocalizations, highlighting the possibility that atypical auditory processing may contribute to impairments in non-verbal social communication.

Atypical auditory processing is a well-documented feature of the autism spectrum. Studies focused on performance-based assessment of auditory processing have identified difficulties in the ability to identify distinct rapidly presented stimuli (i.e., gap detection; (32), temporal processing efficiency (33), and discrimination of rapid sound sequences (34), which are thought to underlie broader language and communication challenges in ASD. (34) reported impairments in frequency discrimination, particularly at high standard frequencies, which measures the ability to perceive a change in the frequency of a pure tone. Impaired auditory temporal integration in autistic children may contribute to less effective integration of multisensory cues, such as auditory and visual cues, into a coherent whole, thereby resulting in difficulties in perceiving speech in noise (for a review see 35). In fact, in addition to basic auditory processing deficits, behavioral studies have shown that individuals with ASD often struggle with perceiving speech in challenging listening environments (36–38). Difficulties emerge in conditions involving competing speech or temporally fluctuating noise, pointing to deficits in temporal processing and attentional control (35, 37). Several studies have found abnormal interaural asymmetries with a left ear deficit on dichotic speech tasks for children on the spectrum (39, 40). Denman et al. (41) also observed that dichotic auditory training up to twelve weeks can improve auditory and language abilities in ASD children. This evidence indicates that impaired auditory processing may contribute to real-world communication challenges, particularly in noisy environments, and suggest that targeted auditory training might improve language abilities during development.

Abnormalities in timing of auditory processing also have been identified at the cortical level using magnetoencephalography (MEG). For instance, individuals with ASD have been reported to demonstrate delayed auditory evoked response components (42–49). Additionally, Edgar et al. (44) found delayed bilateral M50 responses and increased missing M100 responses in ASD children and adolescents compared to neurotypical peers when exposed to binaural tones. Impaired rapid cortical processing of both basic auditory information (42, 50, 51) as well as speech sounds (52) also has been repeatedly demonstrated in individuals with autism.

There is growing evidence that temporal auditory processing dysfunction may contribute to the communication impairments observed in autism. Roberts et al. (49) showed longer latencies of M50 and M100 responses to puretones in minimally verbal and non verbal children compared to those with better verbal communication skills within the autistic population, indicating an association between language abilities and cortical auditory responses. Moreover, mismatch field latencies were prolonged in children on the spectrum compared to neurotypical children during the presentation of tones and vowels, with a more pronounced trend in ASD children with language impairment (47). Matsuzaki et al. (45) also reported delayed mismatch field latencies to vowel stimuli in minimally verbal and nonverbal ASD children, compared to verbal ASD and neurotypical children.

These delays in cortical response to sound are thought to make it difficult to process sounds at the rapid pace necessary for processing speech. Indeed, studies of cortical rapid auditory processing have identified associations between rapid auditory dysfunction and communication abilities. For example, Oram Cardy et al. (51) reported that ASD individuals with language/phonological processing impairment produced less identifiable MEG responses to the second click in a rapid paired-click paradigm compared to ASD individuals with intact language abilities. Rapid processing of paired puretones sounds has been associated with phonological awareness, vocabulary performance, and speech articulation in youth with autism (50). This study also found that phonological processing mediated the relationship between cortical rapid tone processing and overall language abilities in autistic participants, suggesting that inability to reliably process rapidly presented basic sounds may impact the ability to process speech sounds, with downstream effect on language function. This interpretation was supported by a second study demonstrating that cortical rapid processing of paired speech sounds was directly associated with articulation, expressive language and vocabulary, and phonological memory, with phonological memory mediating the association between cortical rapid speech processing and receptive language (52). Collectively, these findings suggest that impairment in rapid processing of speech sounds may impact ability to process the sounds of one’s own speech (necessary for speech production) as well as the ability to hold speech sounds in the phonological working memory, which may impact processing of other’s speech, for some individuals with autism.

Additionally, research suggests that auditory processing difficulties may contribute to impairments in vocal affect recognition, a key component of social interaction. For example, (42) reported that longer M1n response latency and quality of response to the second tone of a rapid paired-tone paradigm was associated with lower vocal affect recognition scores in ASD participants. (25) also found that longer latencies in early perceptual auditory responses were associated with poorer performance on the same vocal affect recognition task. Taken together, these results suggest that impairment in rapid temporal processing might contribute to development of language impairments and non-verbal vocal communication abilities in ASD children.

In sum, prior research on auditory processing in autism suggests that atypical neural responses are closely linked to both verbal and non-verbal communication difficulties. Specifically, converging behavioral and neurophysiological evidence suggests that atypical auditory processing, particularly impairments in temporal resolution and rapid speech processing, can disrupt phonological encoding and working memory mechanisms. These processing deficits, which are observed early during development (16, 53), may influence various aspects of language acquisition, including expressive and receptive skills, speech articulation, and the ability to recognize emotional cues from speech. These social and cognitive abilities are strongly linked to long-term functional outcomes in autistic individuals (54). Therefore, it is important to investigate whether atypical auditory processing development contributes to differences in verbal language abilities and non-verbal vocal communication, which may represent targets for early identification and intervention. While previous studies have established the presence of auditory processing atypicalities and their broad connection to communication challenges, the specific relationship between a range of auditory processing skills and specific verbal and non-verbal communication abilities remains underexplored. Given the importance of auditory processing for social communication, this study aims to explore how differences in auditory processing skills (i.e., gap detection, rapid speech processing, dichotic listening, and speech in noise processing) are associated with measures of both verbal (e.g., expressive and receptive language and vocabulary, speech articulation) and non-verbal communication (e.g., vocal affect recognition). Based on previous findings, we hypothesized that children on the spectrum will exhibit a positive relationship between auditory processing abilities and their performance on verbal and non-verbal communication tasks, potentially shedding light on critical areas for early intervention and support. We predict that auditory processing measures will explain communication skills in the autistic group only, and any associations identified in the neurotypical group will be weak, as both auditory processing and communication abilities are expected to be intact.

## 2 Materials and methods

### 2.1 Participants

Participants were 141 children and adolescents ages 7–17 years. 97 English-speaking participants (59 males, 38 females; age: M = 12.3 years, SD = 2.7) with a DSM-5 (3) diagnosis of ASD and 44 English-speaking neurotypical participants (22 males, 22 females; age: M = 12.3 years, SD = 2.5) took part in the study. Demographic data are shown in **Table 1**. Recruitment targeted individuals on the autism spectrum with both intact and impaired communication skills in an effort to represent a wide range of communication abilities in the sample. Participants with low verbal abilities were included provided they were able to complete the task demands of the measures described below. Inclusion criteria for peripheral hearing were assessed via puretone audiometry, and only participants with valid pure tone thresholds were included. Hearing thresholds at 250Hz, 500Hz, 1000Hz, 2000Hz, 4000Hz, and 8000Hz were measured in dB HL using standard clinical pure-tone audiometry procedures. Air conduction thresholds were obtained monaurally for each ear using a modified Hughson–Westlake procedure, with pulsed tones presented via DD45 headphones using an Amplivox 270+ two-channel diagnostic audiometer. Participants with a three-frequency pure tone average (PTA) greater than 20dB or a hearing threshold greater than 25dB at any individual frequency were excluded.

**Table 1:** Demographics (M ± SD [Range]).

Demographics	ASD individuals (N = 97)	Neurotypical individuals (N = 44)	Z-score/ [t-score]	P-value
Age	12.3 ± 2.7 [7.9-17.4]	12.3 ± 2.5 [8.4-16.8]	[t=0.229]	1.00
Race (N [%])				
Caucasian	53 [55.8%]	20 [45.5%]	1.011	0.3129
Asian	14 [14.2%]	7 [15.9%]	-0.2311	0.8190
African American	3 [3.1%]	0		
Native American	1 [1.1%]	0		
Multiracial	26 [26.8%]	17 [38.6%]	-1.4159	0.161
Ethnicity (N [%])				
Hispanic	23 [23.7%]	6 [13.6%]	1.370	0.1735
Non-Hispanic	74 [76.3%]	38 [86.4%]	-1.370	0.1735
Sex Assigned at Birth (N [%])				
Male	59 [60.8%]	22 [50%]	1.2031	0.2318
Female	38 [39.2%]	22 [50%]	-1.2031	0.2318
Gender Identification (*N* [%])				
Girl/Woman	31 [32%]	22 [50%]	-2.057	**0.043**
Boy/Man	59 [60.8%]	21 [47.7%]	1.455	0.151
Nonbinary	3 [3.1%]	0		
Transgender	3 [3.1%]	1 [2.3%]	0.272	0.7982
Other	1 [1.0%]	0		
Handedness				
Ambidextrous	5 [5.2%]	1 [2.3%]	0.7990	0.4336
Left	11 [11.3%]	4 [9.1%]	0.4071	0.6947
Right	81 [83.5%]	39 [88.6%]	-0.791	0.4325
Pure Tone Average	5.6 ± 8.1 [-10-20]	4.1 ± 6.8 [-10-20]	*[t=1.069]	0.289
SQC Scores	14 ± 7.1 [1-45]	2.7 ± 4.4 [0-28]	*[t=9.743]	**0.001**

Bolded values report the statistically significant differences between groups. SQC scores and gender identification significantly differed between groups.

## 3 Procedures

### 3.1 Diagnostic assessment

The diagnosis of ASD was confirmed based on the DSM-V criteria following a consensus diagnosis from the neuropsychology team under the supervision of licensed clinical psychologists who had achieved research reliability on the Autism Diagnostic Observation Schedule-2nd Edition (ADOS-2) and Autism Diagnostic Interview-Revised (ADI-R) and who made final diagnostic determinations. Diagnostic assignment was informed by information obtained from multiple sources including administration of the ADOS-2, ADI-R, as well as the Social Communication Questionnaire (SCQ; 55) Childhood Autism Rating Scale-2nd Edition (CARS-2; 56), Social Responsiveness Scale-2nd Edition (SRS-2; 57), which were added following implementation of masking mandates due to COVID-19. These autism diagnostic measures were interpreted in the context of additional information provided via a neuropsychological history questionnaire and assessment of language and intelligence via the Clinical Evaluation of Language Fundamentals-Fifth Edition (CELF-5; 58), Wechsler Intelligence Scale for Children-Fifth Edition (WISC-V; 59), and the Test of Nonverbal Intelligence-Fourth Edition (TONI-4; 60). As noted above, while all participants in the ASD group were administered the SCQ, ADOS-2, and ADI-R as part of the diagnostic confirmation evaluation, administration of the ADOS-2 was performed under masking requirements during the COVID-19 pandemic for some study participants, which deviates from the standardized administration that was developed well before the pandemic. Following recommendations from ADOS-2 certified trainers at our site, these ADOS-2 administrations were not scored due to this deviation from standardization, and observational information from the ADOS-2 was used to inform diagnostic determination, along with the addition of alternative measures of autism symptomatology to further inform diagnosis, including the CARS-2 and SRS-2, administered to all participants enrolled after implementation of masking requirements (N = 56).

### 3.2 Verbal communication

Norm-referenced scores measuring receptive and expressive language skills were obtained using the CELF-5) (58). Expressive vocabulary was assessed using the Expressive Vocabulary Test-Third Edition (EVT-3; 61), and receptive vocabulary was evaluated using the Peabody Picture Vocabulary Test-Fifth Edition (PPVT-5; 62). Additionally, articulatory accuracy was measured using the Sounds-In-Words subtest of the Goldman-Fristoe Tests of the Articulation-Third Edition (GFTA-3; 63). All scores were age-scaled norm-referenced scores, with higher values corresponding to better performance.

### 3.3 Non-verbal vocal communication

The ability to recognize emotional cues in speech was evaluated using the Child and Adult Paralanguage subtests of the Diagnostic Analysis of Nonverbal Accuracy-2nd Edition (DANVA-2; 64). This computer-based evaluation measures the ability to identify affective content in identical, semantically neutral statements (e.g., “I’m going out of the room right now, but I’ll be back later”) spoken with different emotional inflections, including happiness, sadness, anger, or fear. Each of these subtests (i.e., child or adult voices) consist of 24 stimuli. Reliability assessment of the DANVA-2 Paralanguage subscales has resulted in a Cronbach’s alpha coefficient of 0.77 and retest reliability of r = .74 at 4 weeks posttest (64). Age-scaled standard scores were derived for each subtest based on the total number of errors, and then a standard score “vocal affect recognition composite” was derived from the average of the two standard scores.

### 3.4 Auditory processing tasks

To assess auditory processing abilities we employed four tests from the age-appropriate (child or adolescent) SCAN-3 Tests for Auditory Processing Disorders (65) test battery, including Gap Detection, Competing Words-Free Recall, Auditory Figure-Ground 8dB, and Time-Compressed Sentences tasks. The Gap Detection task assesses the ability of a person to detect both sounds of a rapid tone pair. Participants were presented with nine pairs of tones separated by varying silent intervals ranging from 0 to 40 milliseconds, in both ears. For each pair, participants reported whether they heard one continuous tone or two separate tones. The number of correctly identified tone pairs were totaled to compute a raw score for each participant, as age-scaled scores are not available for this task. For the Competing Words-Free Recall test, 20 word pairs were presented dichotically, so that two different words are played simultaneously, one in each ear. The participant is asked to repeat both words. The Auditory Figure-Ground (+8 dB) test presents 40 words to either the right or left ear at an intensity of 8dB louder than a multispeaker speech background noise. Participants were required to repeat each of these 40 words. The Time-Compressed Sentences test involved presenting 20 short sentences at an accelerated speech rate. Participants were asked to repeat each entire sentence, and their performance was scored based on the number of targeted words they correctly repeated. Age-scaled scores were computed and analyzed for the Competing Words, Auditory Figure Ground, and Time Compressed Sentences tasks and raw scores were analyzed for the Gap Detection task.

### 3.5 Data analysis plan

To examine the association between auditory processing abilities and both verbal and non-verbal communication skills, we conducted six hierarchical regression analyses, one for each of the measures of auditory communication. For each of the analyses, we controlled for age and intellectual abilities by entering TONI-4 nonverbal IQ scores and age in step 1. Then all auditory processing measures were entered in step 2 to determine whether the linear combination of auditory processing measures was significantly associated with each communication variable after controlling for age and nonverbal intelligence. The Benjamini–Hochberg (BH) correction for multiple comparisons was applied to control the false discovery rate (FDR), with an adjusted significance threshold of 0.05. Data visualization was performed in R (66) using the ggplot2 package (67).

## 4 Results

Descriptive statistics, including means, standard deviations for each of the main variables are shown in **Table 2**. **Table 3** presents the results of the hierarchical linear regression analyses. Following Benjamini-Hochberg correction for six comparisons (*q* <.05), five out of six regression analyses remained statistically significant.

**Table 2:** Descriptive statistics for auditory processing and communication variables (M ± SD [Range]).

Communication Variables	ASD individuals	Neurotypical individuals	t-value	p-value
Receptive language (CELF-5)	101 ± 21.1 [50-141]	113.5 ± 9.9 [92-133]	−5.09	<0.001
Expressive Language (CELF-5)	102.4 ± 20.4 [45-139]	113.7 ± 12.0 [85-135]	−4.35	<0.001
Receptive Vocabulary (PPVT-5)	108.2 ± 22.9 [46-160]	117.9 ± 14.4 [83-149]	−3.22	0.001
Expressive Vocabulary (EVT-3)	109.2± 22.7 [57-160]	117.9 ± 13.2 [83-147]	−3.03	0.003
Articulation (GFTA-3)	86 ± 24.7 [39-109]	102.3 ± 5.1 [84-109]	−6.68	<0.001
Non-verbal communication (DANVA-2)	96 ± 14.2 [38-123]	104.8 ± 11 [85.5-121]	−4.21	<0.001
Gap detection	7 ± 2.1 [1-9]	7.8 ± 1.2 [5-9]	−3.03	0.003
Competing words-free recall	8.7 ± 2.8 [1-15]	11.0 ± 2.3 [5-16]	−5.39	<0.001
Time-compressed sentences	9.8 ± 3.1 [1-16]	10.5 ± 2.4 [6-16]	−1.54	0.127
Auditory figure-ground 8dB	9.1 ± 3.0 [1-14]	10.5 ± 2.1 [6-14]	−3.36	0.001

**Table 3:** Hierarchical regression analyses examining variance in communication skills explained by auditory processing measures in autistic (ASD) and neurotypical (TDC) youth.

Model	ASD				TDC			
	ΔR^2^	β	b [95% CI]	p	ΔR^2^	β	b [95% CI]	p
Receptive Language (CELF-5)								
**Step 1**	.301***				**.181**			
Age		-.016	-0.13 [-1.53, 1.28]	.860		.108	0.42 [-0.90, 1.74]	.523
Nonverbal IQ		.401***	**0.67 [0.37, 0.96]**	<.001		.493**	0.55 [0.17, 0.93]	.006
**Step 2**	.157***				**.112**			
Gap Detection		.272**	**2.76 [0.98, 4.54]**	.003		.110	0.91 [-1.65, 3.46]	.477
Competing Words Free Recall		.056	0.41 [-1.01, 1.83]	.564		.126	0.55 [-0.83, 1.93]	.424
Time-Compressed Sentences		.266**	**1.85 [0.50, 3.20]**	.008		.153	0.64 [-0.71, 1.99]	.341
Auditory Figure-Ground 8dB		-.020	-0.14 [-1.36, 1.08]	.816		.228	1.08 [-0.50, 2.65]	.174
**Total R^2^**		.458***				.293		
Expressive Language (CELF-5)								
**Step 1**	.255***				**.043**			
Age		.091	0.70 [-0.68, 2.09]	.316		.159	0.75 [-0.97, 2.47]	.385
Nonverbal IQ		.394***	0.63 [0.34, 0.92]	<.001		.166	0.23 [-0.27, 0.72]	.364
**Step 2**	.181***				**.136**			
Gap Detection		.191*	1.87 [0.12, 3.62]	.037		.260	2.59 [-0.74, 5.92]	.124
Competing Words Free Recall		.039	0.28 [-1.12, 1.67]	.696		.280	1.49 [-0.31, 3.28]	.102
Time-Compressed Sentences		.368***	**2.47 [1.14, 3.80]**	<.001		-.194	-0.98 [-2.74, 0.78]	.265
Auditory Figure-Ground 8dB		-.018	-0.13 [-1.33, 1.08]	.836		.010	0.06 [-1.99, 2.11]	.954
**Total R^2^**		.436***				.179		
Receptive Vocabulary (PPVT-5)								
**Step 1**	.224***				.351***			
Age		-.085	-0.70 [-2.25, 0.85]	.371		-.206	-1.17 [-2.90, 0.56]	.180
Nonverbal IQ		.306**	0.53 [0.20, 0.85]	.002		.558***	**0.91 [0.41, 1.41]**	<.001
**Step 2**	.168***				**.076**			
Gap Detection		.218*	2.29 [0.34, 4.25]	.022		-.013	-0.15 [-3.51, 3.20]	.926
Competing Words Free Recall		.026	0.20 [-1.37, 1.76]	.804		.076	0.49 [-1.32, 2.30]	.589
Time-Compressed Sentences		.350**	**2.53 [1.05, 4.02]**	.001		.260	1.59 [-0.19, 3.36]	.078
Auditory Figure-Ground 8dB		-.040	-0.29 [-1.63, 1.05]	.666		.002	0.02 [-2.05, 2.09]	.987
**Total R^2^**		.392***				.427		
Expressive Vocabulary (EVT-3)								
**Step 1**	.261***				**.107**			
Age		.100	0.85 [-0.81, 2.51]	.310		.220	1.33 [-0.53, 3.19]	.181
Nonverbal IQ		.443***	**0.78 [0.43, 1.13]**	<.001		.339*	0.72 [0.02, 1.25]	.042
**Step 2**	**.074**				**.105**			
Gap Detection		.142	1.54 [-0.56, 3.64]	.148		.160	1.75 [-1.85, 5.35]	.331
Competing Words Free Recall		.103	0.81 [-0.87, 2.48]	.340		.200	1.17 [-0.77, 3.11]	.230
Time-Compressed Sentences		.174	1.29 [-0.30, 2.88]	.111		.163	0.91 [-0.99, 2.81]	.338
Auditory Figure-Ground 8dB		-.054	-0.41 [-1.85, 1.03]	.576		-.061	-0.39 [-2.60, 1.84]	.727
**Total R^2^**		.336***				.212		
Articulation (GFTA-3)								
**Step 1**	.070*				**.037**			
Age		.068	0.62 [-1.33, 2.56]	.530		-.210	-0.42 [-1.19, 0.36]	.282
Nonverbal IQ		.219*	0.41 [0.01, 0.82]	.047		-.059	-0.03 [-0.26, 0.19]	.762
**Step 2**	.132**				**.030**			
Gap Detection		-.013	-0.15 [-2.60, 2.31]	.905		-.144	-0.61 [-2.11, 0.90]	.419
Competing Words Free Recall		.073	0.61 [-1.35, 2.57]	.538		-.130	-0.29 [-1.10, 0.52]	.469
Time-Compressed Sentences		.123	0.97 [-0.89, 2.83]	.303		.062	0.13 [-0.66, 0.93]	.736
Auditory Figure-Ground 8dB		.270*	2.16 [0.48, 3.85]	.012		.098	0.24 [-0.69, 1.16]	.606
**Total R^2^**		.202**				.068		
Non-verbal Comm. (DANVA-2)								
**Step 1**	**.060**				**.090**			
Age		-.089	-0.48 [-1.62, 0.67]	.410		.066	0.21 [-0.96, 1.37]	.723
Nonverbal IQ		.102	0.11 [-0.13, 0.35]	.353		.255	0.23 [-0.11, 0.56]	.181
**Step 2**	.160**				**.058**			
Gap Detection		.099	0.67 [-0.76, 2.10]	.357		-.057	-0.38 [-2.64, 1.88]	.737
Competing Words Free Recall		-.141	-0.69 [-1.84, 0.46]	.233		.063	0.22 [-1.02, 1.45]	.723
Time-Compressed Sentences		.427***	**1.97 [0.89, 3.05]**	<.001		.004	0.01 [-1.23, 1.25]	.984
Auditory Figure-Ground 8dB		.068	0.31 [-0.66, 1.29]	.525		.264	0.99 [-0.40, 2.38]	.158
**Total R^2^**		.220**				.148		

β, standardized beta coefficient from the final model (Step 2). b, unstandardized beta coefficient with 95% confidence intervals. ΔR^2^, R-squared change at each step. A Bonferroni correction for multiple comparisons across the six models set the significance threshold at *p* <.0083. Independent variables that remain significant after this correction are shown in bold.

ASD, Autism Spectrum Disorder; TDC, Typically Developing Children; CELF-5, Clinical Evaluation of Language Fundamentals- Fifth Edition; PPVT-5, Peabody Picture Vocabulary Test- Fifth Edition; EVT-3, Expressive Vocabulary Test- Third Edition; GFTA-3, Goldman-Fristoe Test of Articulation- Third Edition; DANVA-2, Diagnostic Analysis of Nonverbal Accuracy- 2nd Edition.

p <.05. **p <.01. ***p <.001.

### 4.1 Receptive language (CELF-5)

**Figure 1:**
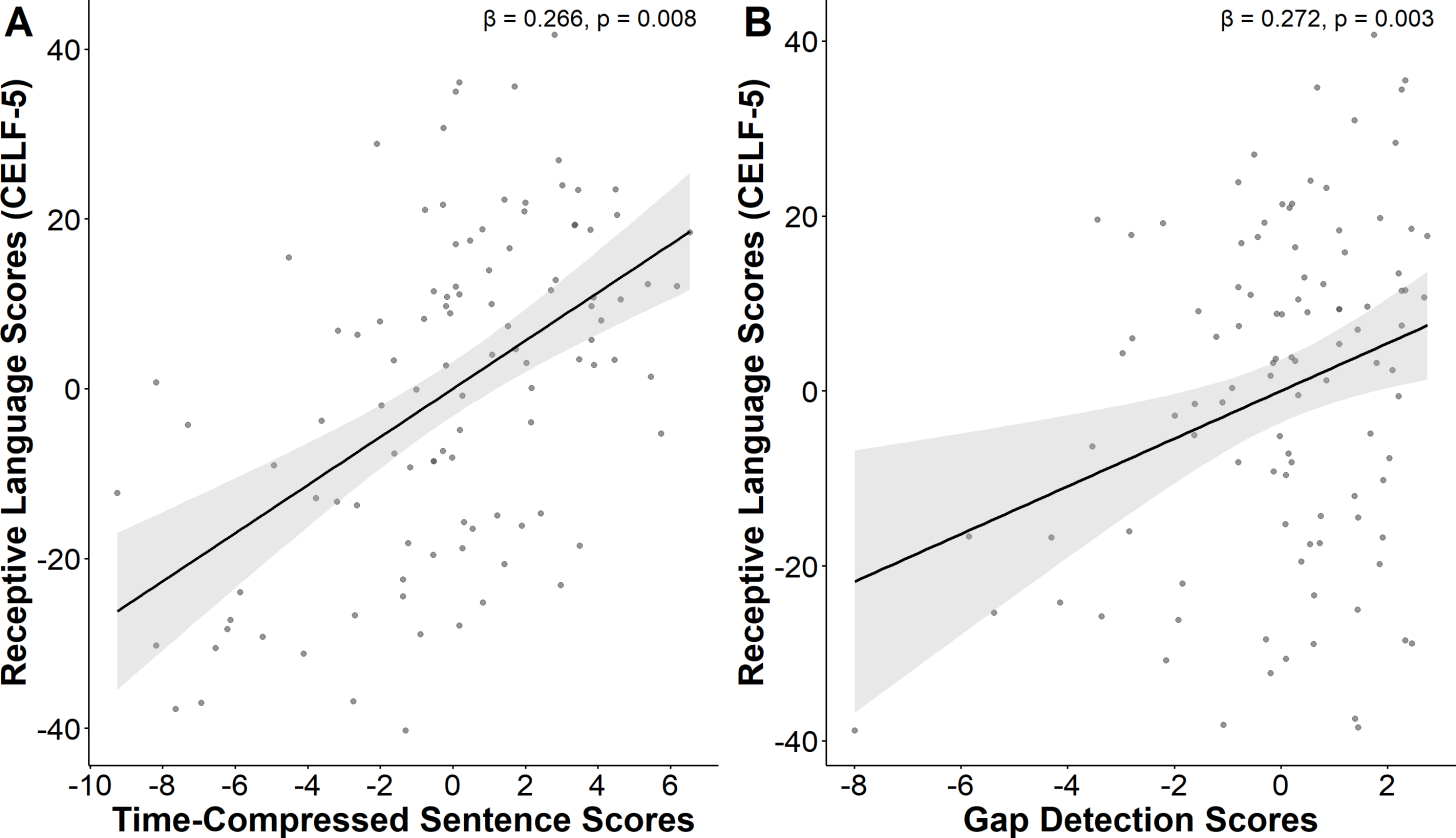
Receptive language partial correlation plots. positive associations were identified between receptive language scores and **(A)** time compressed sentence scores and **(B)** gap detection scores **(B)**, after controlling for non-verbal IQ and age in autistic individuals.

Step 1 of the hierarchical regression analysis within the ASD group indicated that the linear combination of age and nonverbal IQ accounted for 30.1% of the variance in receptive language, with F(2,91)=19.619, ΔR^2^ = 0.301, p<0.001. Statistical tests on beta weights indicated that only nonverbal IQ was significantly associated with receptive language. In Step 2, auditory processing measures were significantly associated with the CELF-5 receptive language index scores, accounting for 15.7% of the variance after controlling for age and non-verbal IQ (F(4, 87) = 6.295, ΔR^2^ = 0.157, *p* < 0.001). Specifically, performance on the Gap Detection and Time-Compressed Sentences tasks were associated with better receptive language outcomes (**Figure 1**). In contrast, Auditory Figure-Ground and Competing Words were not significantly associated with receptive language. In the neurotypical group, Step 1 in the hierarchical regression was statistically significant, with F(2,40)=4.419, ΔR^2^ = 0.181, p=0.018, indicating that the linear combination of age and nonverbal IQ accounted for 18.1% of the variance in receptive language. Statistical tests on beta weights indicated that only nonverbal IQ was significantly associated with receptive language. In Step 2, no significant effect was found between any auditory processing measures and receptive language skills (**Table 3**).

### 4.2 Expressive language (CELF-5)

**Figure 2:**
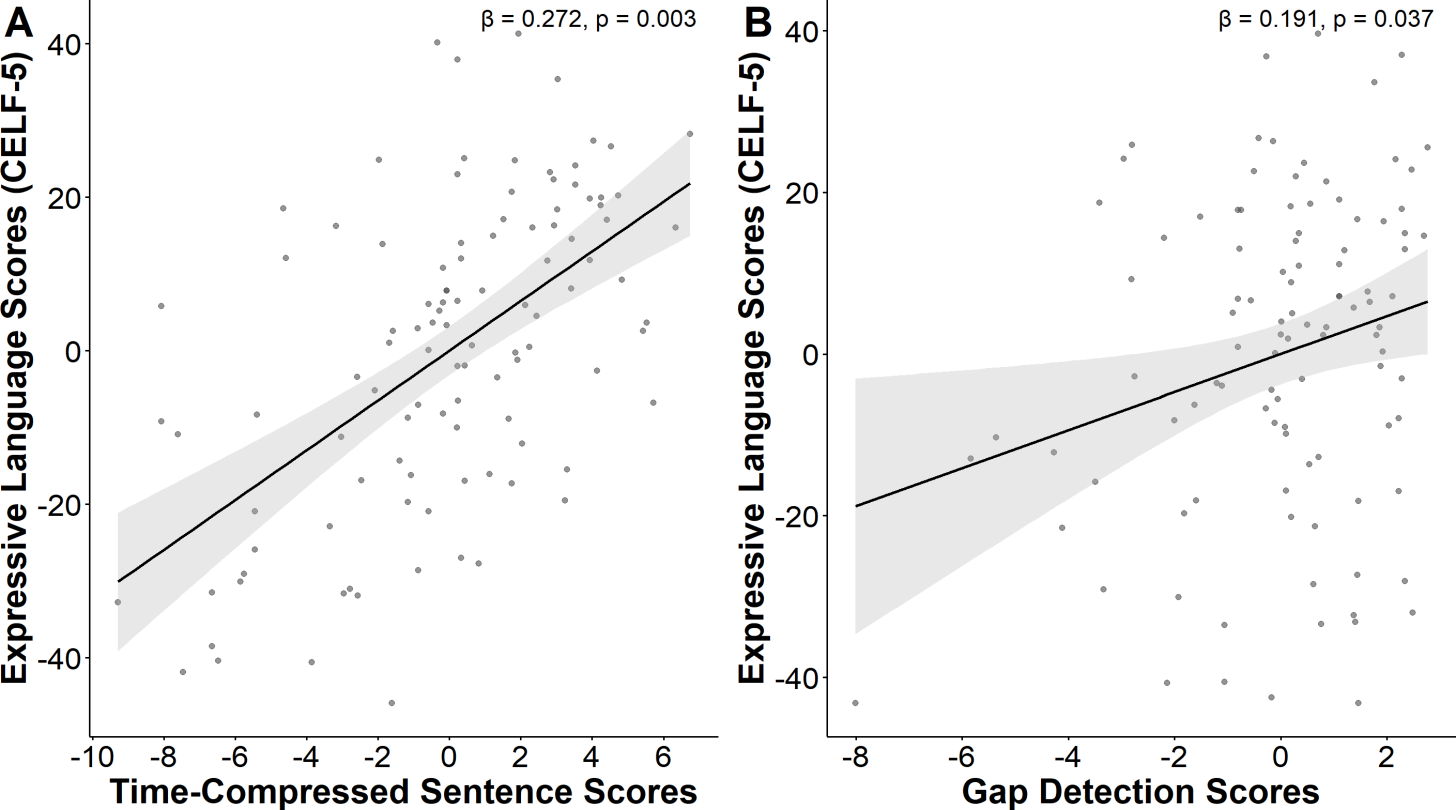
Expressive language partial correlation plots. positive associations were identified between expressive language scores and **(A)** time compressed sentence scores and **(B)** gap detection scores **(B)**, after controlling for non-verbal IQ and age in autistic individuals.

Step 1 of the hierarchical regression analysis within the ASD group indicated that the linear combination of age and nonverbal IQ accounted for 23.9% of the variance in expressive language, with F(2,91)=15.566, ΔR^2^ = 0.239, p<0.001. Statistical tests on beta weights indicated that only nonverbal IQ was significantly associated with expressive language. In Step 2, auditory processing measures were significantly linked to the CELF-5 expressive language index scores in autistic children, after controlling for non-verbal IQ scores (F(4, 87) = 6.966, ΔR^2^ = 0.181, p < 0.001). Gap detection and Time-Compressed Sentences performance was positively associated with expressive language abilities (**Figure 2**). Auditory Figure-ground and Competing Words were not significantly associated with expressive language. In the neurotypical group, neither Step 1 and 2 in the hierarchical regression were statistically significant (**Table 3**).

### 4.3 Receptive vocabulary (PPVT-5)

**Figure 3:**
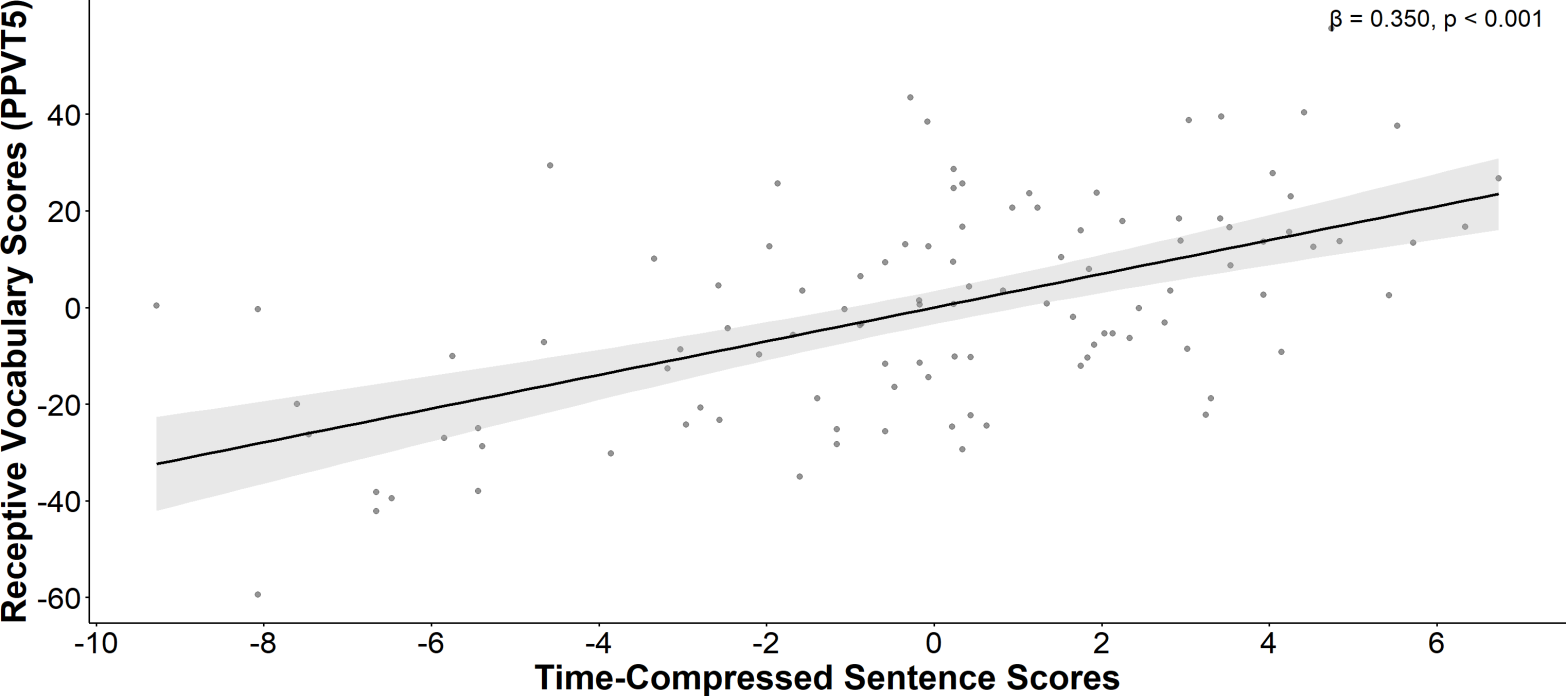
Receptive vocabulary partial correlation plot. A positive association was identified between Time Compressed Sentence scores and receptive vocabulary scores, after controlling for non-verbal IQ and age in autistic individuals.

Step 1 of the hierarchical regression analysis within the ASD group indicated that the linear combination of age and nonverbal IQ accounted for 20.7% of the variance in receptive vocabulary, with F(2,91)=13.113, ΔR^2^ = 0.207, p<0.001. Statistical tests on beta weights indicated that only nonverbal IQ was significantly associated with receptive vocabulary. In Step 2, auditory processing abilities were also significantly associated with receptive vocabulary scores on the PPVT-5, explaining 16.8% of the variance after adjusting for non-verbal IQ scores (*F*(4, 87) = 6.008, Δ*R^2^* = 0.168, *p* < 0.001). Gap detection and Time-compressed sentences abilities emerged again as the only variables significantly associated with receptive vocabulary scores (**Figure 3**). In the neurotypical group, Step 1 in the hierarchical regression indicated that the linear combination of age and nonverbal IQ accounted for 35.1% of the variance in receptive vocabulary, with F(2,40)=10.795, ΔR^2^ = 0.351, p<0.001. Statistical tests on beta weights indicated that only nonverbal IQ was significantly associated with receptive language. In Step 2, no significant effect was found between any auditory processing measures and receptive vocabulary skills (**Table 3**).

### 4.4 Expressive vocabulary (EVT-3)

Step 1 of the hierarchical regression analysis within the ASD group indicated that the linear combination of age and nonverbal IQ accounted for 24.5% of the variance in expressive vocabulary, with F(2,91)=16.092, ΔR^2^ = 0.245, p<0.001. Statistical tests on beta weights indicated that only nonverbal IQ was significantly associated with expressive vocabulary. Step 2 was not statistically significant, with F(2,91)=2.435, ΔR^2^ = 0.074, p<0.053, indicating that the linear combination of auditory processing measures were not significantly associated with expressive vocabulary scores. No significant effects were identified in the neurotypical group.

### 4.5 Articulation (GFTA-3)

**Figure 4:**
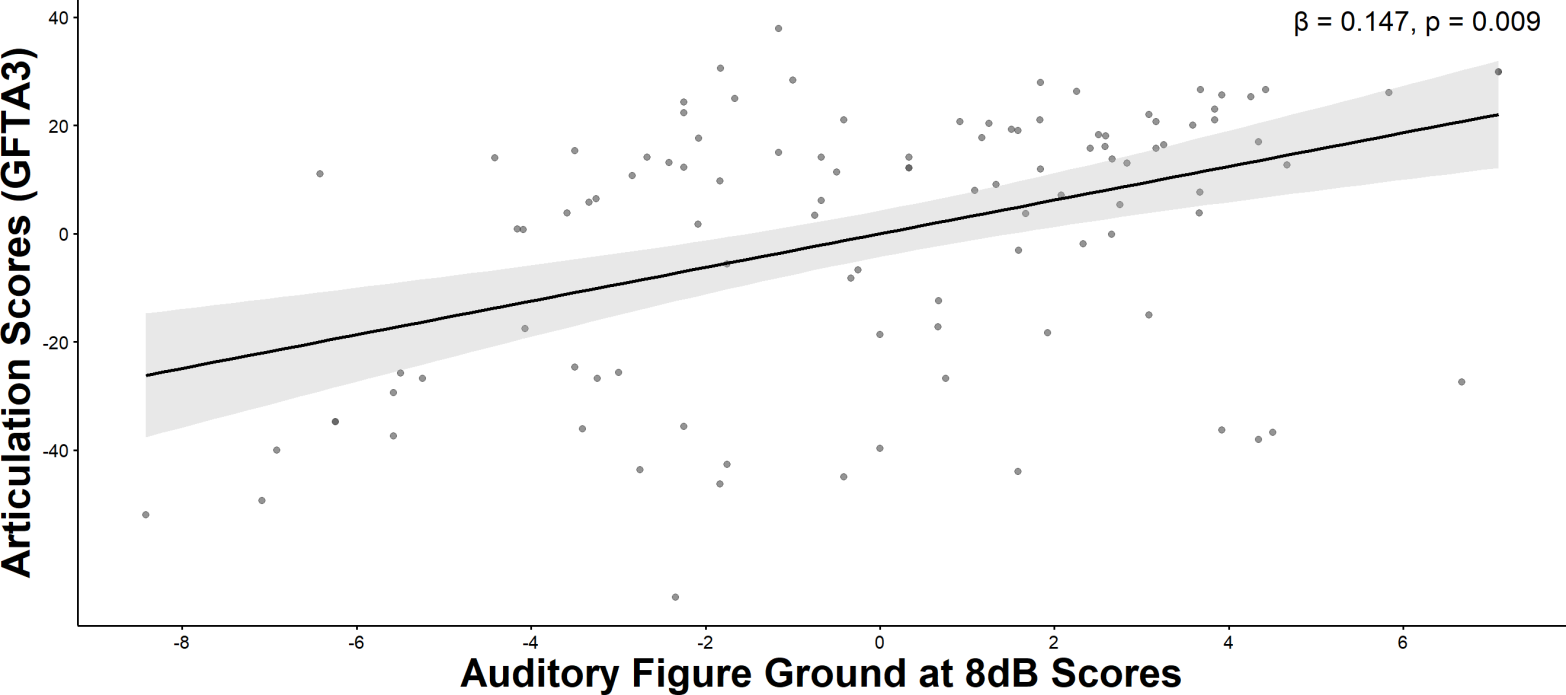
Articulation partial correlation plot. A positive association was identified between Auditory Figure Ground at 8dB scores and articulation scores, after controlling for non-verbal IQ and age in autistic individuals.

Step 1 of the hierarchical regression analysis within the ASD group indicated that the linear combination of age and nonverbal IQ accounted for 5% of the variance in articulation, with F(2,91)=3.430, ΔR^2^ = 0.050, p<0.037. Statistical tests on beta weights indicated that only nonverbal IQ was significantly associated with articulation. In Step 2, auditory processing measures accounted for 14.7% of the variance in articulation scores on the GFTA-3 after controlling for non-verbal IQ (*F*(4, 87) = 3.604, Δ*R^2^* = 0.132, *p* = 0.009). Only Auditory Figure-Ground was significantly associated with articulation performance (**Table 3**, **Figure 4**). In the neurotypical group, neither Step 1 nor Step 2 were statistically significant.

### 4.4 Non-verbal communication (DANVA-2)

**Figure 5:**
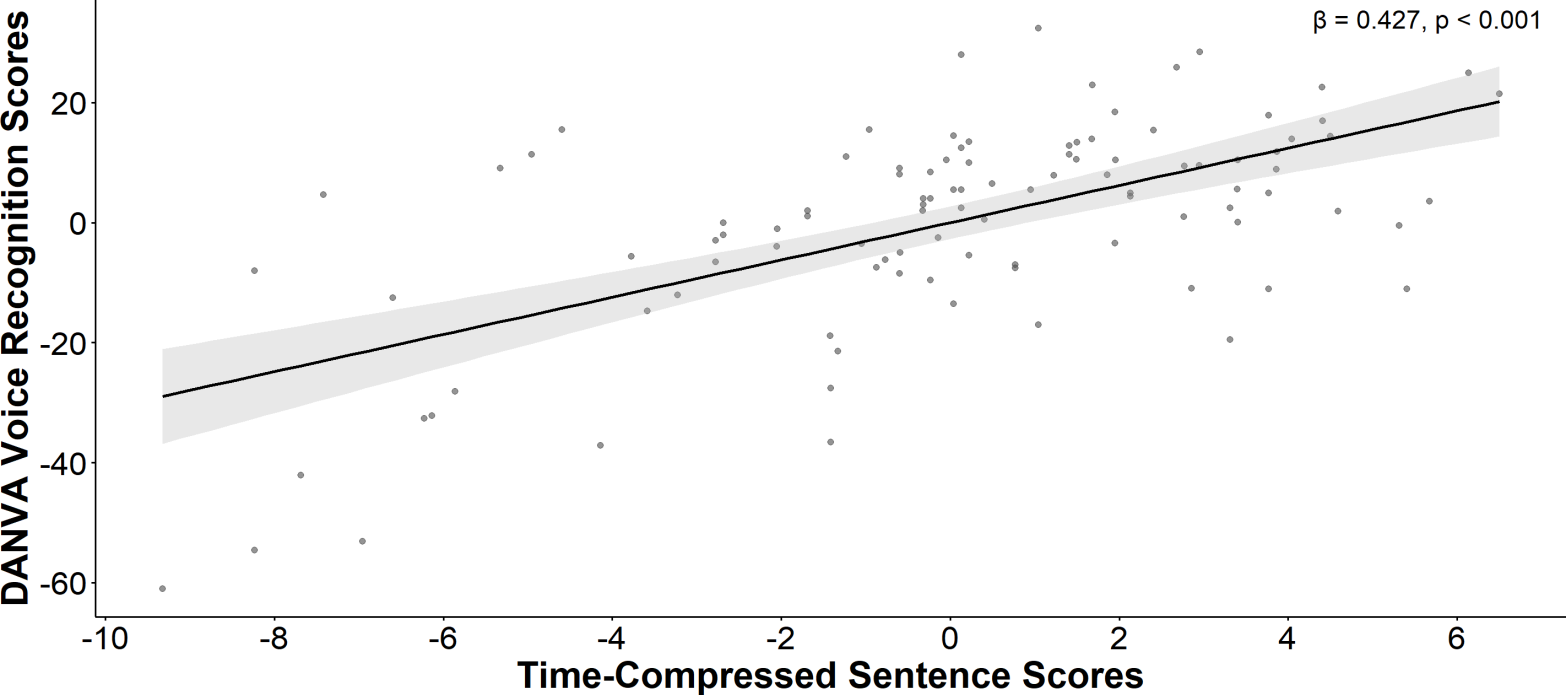
Vocal affect recognition [artial correlation plot. A positive association was identified between Time-Compressed Sentence scores and vocal affect recognition scores, after controlling for non-verbal IQ and age in autistic individuals.

Step 1 of the hierarchical regression analysis within the ASD group indicated that neither nonverbal IQ nor age were significantly associated with vocal affect recognition. In Step 2, vocal affect recognition ability was significantly associated with the auditory processing measures (*F*(4, 85) = 4.359, Δ*R^2^* = 0.165, *p* = 0.003). Among the independent variables, only the Time-Compressed Sentences task showed a significant positive association with vocal affect recognition composite scores (**Table 3**, **Figure 5**). In the neurotypical group, neither Step 1 nor Step 2 were statistically significant.

## 5 Discussion

The current study investigated the relationships between auditory processing performance and verbal and non-verbal vocal communication abilities in autistic and neurotypical children. In autistic children, auditory processing measures were linked to performance in verbal and non-verbal vocal communication. While we did not observe significant associations between auditory processing and communication abilities in the neurotypical group, this finding should be interpreted cautiously given the smaller sample size, as analyses were only powered to detect large effects. Effect sizes in the neurotypical group were small to moderate, suggesting weaker associations than those observed in the autistic group. This is to be expected given that the neurotypical group is expected to have reduced variability in both auditory processing and communication skills (i.e., more likely to be in the average range). However, we cannot exclude the possibility that the absence of significant effects reflects limited power and generalizability from our small sample of neurotypical participants rather than true group differences. The results do, however, give a clearer picture of the complex interplay between auditory processing and communication abilities in children on the spectrum.

### 5.1 Verbal communication

While age was not significantly associated with performance on measures of verbal communication, nonverbal IQ was significantly associated with receptive language, as well as receptive and expressive vocabulary scores, in both groups. However, nonverbal IQ was not associated with expressive language and articulation scores in the neurotypical group, and it was not associated with vocal affect recognition abilities in either group.This is consistent with previous research demonstrating associations between nonverbal IQ and both receptive and expressive language (68).

As hypothesized, auditory processing measures were associated with verbal communication skills in children with ASD. Specifically, Time-Compressed Sentences scores (i.e., the ability to comprehend and accurately repeat speech presented at an accelerated rate) were associated with both expressive and receptive language and receptive vocabulary skills. Notably, the Time Compressed Sentences task involves both rapid speech processing to perceive the sentence and working memory to mentally maintain the verbal information long enough to repeat it. These findings are consistent with our previous MEG study in which we identified a direct relationship between cortical rapid processing of speech sounds and expressive language and vocabulary and an indirect association with receptive language, by way of a mediating effect of phonological memory (50). Gap detection scores also were found to be associated with receptive and expressive language skills. This suggests that temporal resolution abilities, as reflected in gap detection performance, may support a broad range of linguistic processes, consistent with prior research demonstrating associations between cortical rapid tone processing and verbal communication skills (50, 51). Therefore, efficient temporal auditory processing likely contributes to the precise analysis of rapid acoustic cues that are important not only for perceiving speech but also for monitoring and refining speech output. While receptive language tasks place clear demands on the rapid analysis of auditory input, expressive language also relies on accurate temporal representations to support phonological encoding and speech planning. Although auditory feedback is not strictly necessary for speech production, it plays a role in maintaining speech accuracy and fluency. There is also a large body of research demonstrating associations between delays in basic cortical auditory response latency and language impairment in autism (43, 45–47, 69–72). These may suggest that sluggish cortical auditory responses provide insufficient access to sound processing at the rapid pace necessary for interpreting speech. This prior work demonstrates a potential causal path by which inefficient sound processing impacts the component skills that are necessary for language. The present study further supports this hypothesis by demonstrating a direct association between rapid sound and sentence processing skills and language abilities in autistic youth, providing novel performance-based evidence of the neurobiological associations between rapid processing and language reported in these previous studies.

Our findings also reveal a robust association between articulation accuracy and speech-in-noise performance, suggesting that the ability to perceive relevant sounds accurately is crucial for precise speech sound production. This aligns with several models of speech production that suggest the role of auditory feedback and sensorimotor integration in guiding and fine-tuning articulatory movements (69, 73–78). When listeners effectively segregate speech from competing noise, they receive clearer phonetic cues that help establish and maintain precise internal models of speech (79). Complementing this sensorimotor perspective, the motor theory of speech perception suggests that perceivers interpret acoustic signals by mapping them onto the articulatory gestures that produce them (80, 81). Intracranial and neuroimaging studies support this theory, showing that listening to speech activates motor and premotor regions (82–84). Responses in the motor cortex during speech perception are found to be organized along acoustic features in a manner similar to auditory cortex (82), suggesting that auditory representations are encoded within motor regions. Consequently, poorer abilities to accurately perceive and repeat target words presented among background noise may impair the development of speech sound perception, potentially affecting articulation accuracy in autistic individuals. Indeed, disruptions in the auditory-motor feedback loop essential for real-time speech monitoring have been demonstrated in 16p11.2 deletion syndrome, the most common genetic etiology of autism which is strongly associated with speech impairment (85). Simarro Gonzalez et al. (86) found that better articulation accuracy and language abilities were associated with stronger fine motor skills, such as manual dexterity, visual-motor integration, and pencil control, suggesting a link between motor coordination and abilities and spoken language outcomes. Together, these findings indicate that the well-established dependency of effective speech production on both accurate auditory perception and the integration of sensory input with motor control may be disrupted in ASD individuals that present impaired language and communication abilities.

### 5.2 Non-verbal vocal communication

Our results also revealed a close relationship between auditory processing and non-verbal communication skills, particularly in the recognition of vocal affective cues. We observed a strong association between Time-Compressed Sentences scores and Vocal Affect Recognition composite scores, suggesting that deficits in rapid speech processing and phonological working memory may impair the ability to interpret emotional information conveyed through acoustic vocal cues. These findings are consistent with prior research suggesting that altered sensory information processing may contribute to presentation of social traits in ASD (87, 88), supporting the hypothesis that challenges in recognizing vocal emotion in autism may be due to perceptual difficulties (30). Indeed, prior work has demonstrated that cortical auditory response delays and impaired cortical rapid processing were associated with vocal affect recognition performance in autistic youth (25, 42).

Overall, these results add to the growing body of literature suggesting that timely processing of auditory information is necessary for skills in auditory forms of communication, and that inefficient processing may contribute to both verbal and nonverbal communication impairments in autism. Notably, our data also demonstrates that timely processing, while necessary, is not sufficient. Specifically, **Figure 1A** shows that those with impaired gap detection performance consistently had lower language scores, whereas intact gap detection was associated with a range of language functioning. This is to be expected, as basic auditory processing affects spoken language processing at the lowest level, but from there, higher order processes are also necessary in support of language function. These findings further support the hypothesis that temporal limitations in the cortical auditory processing system may have downstream impact on the processing of sound in ways that impact communication for some people with autism. If these cortical delays are present in the early developmental period, they may play a crucial role in the development and refinement of both language comprehension and production, as well as nonverbal communication, in children on the spectrum.

## 6 Limitations and future directions

While this study provides valuable insights into the relationship between auditory processing and verbal and non-verbal vocal communication abilities in autism, there are several limitations to consider. First, the cross-sectional design limits our ability to make causal inferences about the relationship between auditory processing and communication skills. Longitudinal studies are needed to determine whether improvements in auditory processing lead to subsequent gains in language and non-verbal communication abilities. Second, the heterogeneity of the ASD population must be acknowledged. While many individuals on the spectrum showed associations between auditory speech processing and communication abilities, auditory processing difficulties are not a ubiquitous phenomenon on the autism spectrum. Future studies should explore potential subgroups and individual differences within the ASD population to identify whether different patterns of auditory processing difficulties correspond to distinct communication profiles.

## 7 Conclusion

In conclusion, this study highlights the critical role that auditory processing likely plays in both verbal and non-verbal vocal communication in ASD individuals. Our findings indicate that difficulties in auditory temporal processing are closely related to deficits in expressive and receptive language skills, as well as in the ability to recognize vocal affective cues. In contrast, processing of sound in noise was associated with articulatory accuracy. These novel findings highlight distinct mechanisms by which temporal versus spectral components of sound processing may impact different communication functions for some autistic individuals. Incorporating auditory processing interventions into early support strategies for children with ASD may have potential for enhancing both language development and social communication abilities.

## Data Availability

The raw data supporting the conclusions of this article will be made available by the authors, without undue reservation.
